# Deep Neural Network-Based Novel Mathematical Model for 3D Brain Tumor Segmentation

**DOI:** 10.1155/2022/4271711

**Published:** 2022-08-11

**Authors:** Ajay S. Ladkat, Sunil L. Bangare, Vishal Jagota, Sumaya Sanober, Shehab Mohamed Beram, Kantilal Rane, Bhupesh Kumar Singh

**Affiliations:** ^1^Department of Instrumentation Engineering, Vishwakarma Institute of Technology, Pune, India; ^2^Department of Information Technology, Sinhgad Academy of Engineering, Savitribai Phule Pune University, Pune, India; ^3^Department of Mechanical Engineering, Madanapalle Institute of Technology and Science, Madanapalle, Andhra Pradesh, India; ^4^Prince Sattam Bin Abdul Aziz University, Wadi Aldwassir 1191, Saudi Arabia; ^5^Research Centre for Human-Machine Collaboration (HUMAC), Department of Computing and Information Systems, Sunway University, Kuala Lumpur, Malaysia; ^6^Department of Electronics and Communication Engineering, Koneru Lakshmaiah Education Foundation (Deemed to Be University), Vaddeswaram, Andra Pradesh, India; ^7^Arba Minch Institute of Technology, Arba Minch University, Arba Minch, Ethiopia

## Abstract

The use of multimodal magnetic resonance imaging (MRI) to autonomously segment brain tumors and subregions is critical for accurate and consistent tumor measurement, which can help with detection, care planning, and evaluation. This research is a contribution to the neuroscience research. In the present work, we provide a completely automated brain tumor segmentation method based on a mathematical model and deep neural networks (DNNs). Each slice of the 3D picture is enhanced by the suggested mathematical model, which is then sent through the 3D attention U-Net to provide a tumor segmented output. The study includes a detailed mathematical model for tumor pixel enhancement as well as a 3D attention U-Net to appropriately separate the pixels. On the BraTS 2019 dataset, the suggested system is tested and verified. This proposed work will definitely help for the treatment of the brain tumor patient. The pixel level accuracy for tumor pixel segmentation is 98.90%. The suggested system architecture's outcomes are compared to those of current system designs. This study also examines the suggested system architecture's time complexity on various processing units with neuroscience approach.

## 1. Introduction

Tumors are described as the development of glandular growth in the brain, which can be benign or malignant [1, 2], and they are amongst the deadliest diseases [3–5]. The extra growth of tissues at certain places increases the chances of tumor formation. Those with a brain tumor may encounter the following symptoms or signs. A symptom, such as weariness, nausea, or pain, can only be recognized and explained by the individual experiencing it. A symptom is anything that may be observed and quantified by others, such as a fever, rash, or elevated pulse rate [6, 7]. When signs and symptoms are combined, they can help characterize a medical disease. A person with a brain tumor may not display any of the following signs and symptoms. A medical condition other than a brain tumor might be the source of a symptom or sign [8, 9]. Depending on where they originate and how rapidly they grow, astrocytomas could be fatal. As a result, a neurosurgeon's most important and initial goal is to correctly segment a brain tumor so that suitable therapy may be decided [10]. Though brain tumors can cause a number of issues, they are not cancerous, which means they grow slowly and seldom spread to other regions of the body. They also have more well-defined borders, making surgical excision easier, and they seldom recur after removal [11, 12]. Malignant brain tumors, on the other hand, are malignant, grow rapidly, and can spread to other parts of your brain or central nervous system, offering a life-threatening risk [13, 14]. The improved matching filter also aids in reducing the likelihood of misclassification. Progress in the technology of diagnostic imaging and analytical modelling have resulted in improved diagnosis and treatment [1].

To produce thorough scans of the brain, many imaging methods are utilized. MRI scans produce pictures with varied contrasts and brightness that highlight distinct sections of the brain, allowing tumor subregions to be distinguished. Each MRI sequence is thought to be crucial in identifying various tumor subregions. The ability to segment brain tumors is crucial for statistical tumor characterization, which leads to more accurate diagnosis and better treatment methods and strategies [15]. An MRI of the head is a non-invasive, painless treatment that produces detailed images of your brain and brain stem. In an MRI machine, a magnetic field and radio waves are used to produce the images. This test is also known as a cranial MRI or a brain MRI [16, 17]. CT scans employ X-rays, which emit a radiation. MRI, on the other hand, does not use radiation. Due to this, pregnant women cannot take CT scan. People with implants may not take MRI scan. Deep learning advances have resulted in more precise and reliable segmentation algorithms. CNNs have attained state-of-the-art outcomes in a wide range of computer vision applications [18]. By varying the 2D U-Net, Isensee et al. performed a brain tumor segmentation investigation [19] to 3D [15]. Havaei et al. offered a feedforward CNN construction that integrates local and global data to make brain tumor separation [20]. To achieve brain tumor segmentation, Pereira et al. employed short convolution layers and gray-level technique [21]. Any of the following signs and symptoms may not be present in a person with a brain tumor. A symptom might be caused by a medical issue other than a brain tumor. Astrocytomas can be deadly depending on where they come from and how quickly they develop. Brain tumors are not malignant, despite the fact that they can cause a variety of problems.

To improve the validity of the model segmentation network, Kamnitsas et al. [22] employed conditional random field (CRF) as a preprocessing method. Despite the fact that many deep learning algorithms have produced incredibly precise outcomes in diagnostic imaging processing activities, an interpretation is required to realize. This clarification serves as a link between experts and the algorithm, offering correct forecasts in addition to justifications [23]. The terms “explainability,” “interpretability,” and “transparency” have all been used to characterize how well the model is understood [24–26]. For statistical tumor characterization, the capacity to segment brain tumors is critical.

Furthermore, the added dimension of 3D structures complicates understanding the model's findings in order to develop predictions. Medical specialists are hesitant to believe CNN projections because of their lack of explainability and black-box nature [27]. The degree to which a black-box model's projections can be trusted is a hot issue [28]. Understanding the reasons behind the model's forecasts is crucial to avoid bad treatment outcomes and to have trust in the model's forecasts [29]. Furthermore, interpretability allows users to categorize the patterns discovered primarily by the model and check that they are consistent with medical professionals' domain expertise, increasing end-user trust and reliance on the model's judgments [30].

Two primary types of interpretability techniques are presented in [31]. After the model has been trained using the training sample without being significantly altered, post hoc techniques are utilized, whereas ante hoc or trainable attention entails building interpretability into the model architecture from the start. The most common trend in understandability at the intermediate levels of the model is to see and grasp the information gathering process [32]. To investigate the model's secret knowledge, saliency maps are constructed in visual interpretability. Several visual interpretability methodologies have been developed [33–35], and a thorough research of AI has been done [36–38].

Furthermore, representativeness is critical in the healthcare field since it ensures that medical practitioners can recognize and accept estimates of a neural network (NN) [31]. Incorporating optical explicable into deep learning models for medical image interpretation is a popular method, as demonstrated by LIME [36], GB [39], Grad-CAM [40], and CAM [41]. These methods, despite being aesthetically appealing, rely on gradients and image manipulation as inputs. As a result, they are time-consuming to create and produce less clear visual descriptions in terms of projected class. In addition, the lack of assessment criteria makes evaluating the quality of provided explanations difficult. The most typical movement in understandability at the intermediate layers of the model is to view and comprehend the evidence gathering procedure.

According to the literature, the BT segmentation requires extra attention in order to increase its performance metrics. The classification is done based on result of segmentation. The proposed system approach will be incredibly useful in extracting volume of the tumor. The rest of the paper is organized as follows.  Section 2 looks into the planned scheme's mathematical model in further depth. The suggested technique has been tested on many types of data and has produced good results. The suggested system design is compared against a variety of algorithms, and the findings are presented in Section 3. According to Ladkat et al. [42], to assess the temporal complexity of the proposed system, it is tested on various processors. With a conclusion, Section 4 comes to an end. The authors in [43–50] described their trials with machine learning applications in the medical field. The LRA DNN approaches were developed by Shelke et al. [51]. Wankhede et al. [52,53] performed deep learning experiments on the brain tumor.

## 2. Proposed Methodology

Image dataset is passed through equation (1). The low and high components from the image are extracted from the image. The image(*x*, *y*, *z*) is passed through the transform to get *W*_*ψ*_^*i*^(*j*, *p*, *q*, *r*) as a result.

(1) $$\begin{array}{c} W_{\varphi }\left(j_{0},p,q,r\right)=\frac{1}{\sqrt{MN}}\sum_{x=0}^{M-1N-1}\text{image}\left(x,y,z\right)\varphi_{j=0}\left(x,y,z\right), \end{array}$$

(2) $$\begin{array}{c} W_{\psi }^{i}\left(j,p,q,r\right)=\frac{1}{\sqrt{MN}}\sum_{x=0}^{M=1}\sum_{y=0}^{N-1}\text{image}\left(x,y,z\right)\psi^{i}j_{j,m,n}\left(x,y,z\right),i=\left\{H,V,D\right\}, \end{array}$$

where *j*_0_ is an arbitrary starting scale, *W*_*φ*_(*j*_0_, *p*, *q*, *r*) coefficients define an approximation of image(*x*, *y*, *z*) at scale j_0_*W*_*ψ*_^*i*^(*j*, *p*, *q*, *r*) parameters for the scales *j* > = *j*_0_ are *W*_*φ*_ and *W*_*ψ*_^*i*^, image(*x*, *y*, *z*).

(3) $$\begin{array}{c} \text{image}\left(x,y,z\right)=\frac{1}{\sqrt{MN}}\sum_{m}\sum_{n}W_{\varphi }\left(j_{0},p,q,r\right)\varphi_{j_{0},m,n}\left(x,y,z\right)\\ +\frac{1}{\sqrt{MN}}\sum_{i=H,V,D}\sum_{j=j_{0}}^{\infty }\sum_{p}\sum_{q}W_{\psi }^{i}\left(j,p,q,r\right)\psi_{j,p,q,r}^{i}\left(x,y,z\right). \end{array}$$

Now, to get reduced dimensionality features, these decomposed values of the uploaded picture are processed via the following equations:

(4) $$\begin{array}{c} w_{\left(1\right)}=\arg\underset{\left|\left|w\right|\right|=1}{\max}\left\{\left.\sum_{i}{\left(t1\right)}_{\left(i\right)}^{2}\right\}\right.=\arg\underset{\left|\left|w\right|\right|=1}{\max}\left\{\sum_{i}{\left(x_{\left(i\right)}.w\right)}^{2}\right\}, \end{array}$$

(5) $$\begin{array}{c} w_{\left(1\right)}=\arg\underset{\left|\left|w\right|\right|=1}{\max}\left\{\left.{\left\|Xw\right\|}^{2}\right\}\right.=\arg\underset{\left|\left|w\right|\right|=1}{\max}\left\{w^{T}X^{T}Xw\right\}, \end{array}$$

(6) $$\begin{array}{c} w_{\left(1\right)}=\arg\max\left\{\frac{w^{T}X^{T}Xw}{W^{T}W}\right\}. \end{array}$$

Equations (5) and (6) gives the feature and to get the *i*^th^ component of the feature, we need to subtract the first.

(7) $$\begin{array}{c} {\hat{X}}_{i}=X-\sum_{s=1}^{i-1}Xw_{\left(i\right)}w_{\left(i\right)}^{T}, \end{array}$$

(8) $$\begin{array}{c} w_{\left(i\right)}=\arg\underset{\left|\left|w\right|\right|=1}{\max}\left\{{\left\|{\hat{X}}_{k}w\right\|}^{2}\right\}=\arg\max\left\{\frac{w^{T}{\hat{X}}_{i}^{T}{\hat{X}}_{i}\;w}{w^{T}w}\right\}. \end{array}$$

Covariance of the feature set is calculated as

(9) $$\begin{array}{c} Q\left(PC_{\left(j\right),}PC_{\left(k\right)}\right)\propto {\left(Xw_{\left(j\right)}\right)}^{T}\left(Xw_{\left(k\right)}\right)\\ =w_{\left(j\right)}^{T}X^{T}Xw_{\left(k\right)}\\ =w_{\left(j\right)}^{T}\lambda_{\left(k\right)}w_{\left(k\right)}\\ =\lambda_{\left(k\right)}w_{\left(j\right)}^{T}w_{\left(k\right)}. \end{array}$$

Now, equation (8) is supplied as input to the neural network. Equation (9) gives the features which are then fed to the neural network to get the classification done. The all *x*_*ij*_^*ℓ*^expressions in which *ω*_*abc*_occurs can be given as.

(10) $$\begin{array}{c} \frac{\partial E}{\partial \omega_{abc}}=\sum_{i=0}^{N-m}\sum_{j=0}^{N-m}\sum_{k=0}^{N-m}\frac{\partial E}{\partial x_{ij}^{\ell }}\frac{\partial x_{ijk}^{\ell }}{\partial \omega_{abc}}=\sum_{i=0}^{N-m}\sum_{j=0}^{N-m}\sum_{k=0}^{N-m}\frac{\partial E}{\partial x_{ijk}^{\ell }}y_{\left(i+a\right)\left(j+b\right)\left(\text{k}+\text{c}\right)}^{\ell -1}, \end{array}$$

where (*∂x*_*ijk*_^*ℓ*^/*∂ω*_*abc*_) = *y*_(*i* + *a*)(*j* + *b*)(*k* + *c*)_^*ℓ*−1^, which is used as forward propagation equation.

To compute the gradient, there is a need to get the values of *∂E*/*∂x*_*ijk*_^*ℓ*^ where chain rule can be given as

(11) $$\begin{array}{cc} \frac{\partial E}{\partial x_{ijk}^{\ell }}=\frac{\partial E}{\partial y_{ijk}^{\ell }}\frac{\partial y_{ijk}^{\ell }}{\partial x_{ijk}^{\ell }}=\frac{\partial E}{\partial y_{ijk}^{\ell }}\frac{\partial }{\partial x_{ijk}^{\ell }}\left(\sigma \left(x_{ijk}^{\ell }\right)\right)=\frac{\partial E}{\partial y_{ijk}^{\ell }} & \sigma^{\prime }\left(x_{ijk}^{\ell }\right). \end{array}$$

So, it is clear that the error at the current layer *∂E*/*∂y*_*ijk*_^*ℓ*^ can be easily computed by using deltas *∂E*/*∂x*_*ijk*_^*ℓ*^ at the current layer by just using the derivative of the activation function, *σ*′(*x*). We now have everything we need to compute the gradient with regard to the weights employed by this convolutional layer because we know the errors at the current layer. We need to transmit errors back to the preceding layer to compute the weights for present convolutional layer.

(12) $$\begin{array}{c} \frac{\partial E}{\partial y_{ijk}^{\ell -1}}=\sum_{a=0}^{m-1}\sum_{b=0}^{m-1}\sum_{c=0}^{m-1}\frac{\partial E}{\partial x_{\left(i-a\right)\left(j-b\right)\left(\text{k}-\text{c}\right)}^{\ell }}\frac{\partial x_{\left(i-a\right)\left(j-b\right)\left(\text{k}-\text{c}\right)}^{\ell }}{\partial y_{ijk}^{\ell -1}}=\sum_{a=0}^{m-1}\sum_{b=0}^{m-1}\sum_{c=0}^{m-1}\frac{\partial E}{\partial x_{\left(i-a\right)\left(j-b\right)\left(\text{k}-\text{c}\right)}^{\ell }}\omega_{abc}. \end{array}$$

The output of the improved picture is then entered straight into the mathematical model below, which produces enhanced tumor pixels in a three-dimensional plane. Let *R*_*v*_ be the covariance matrix to retrieve all the potential values of the tumor pixels inside the threshold limit.

(13) $$\begin{array}{c} R_{v}=E\left\{vv^{H}\right\}, \end{array}$$

where the conjugate transpose of v is denoted by *v*^*H*^ and *y* represents the many vector permutations that maybe produced from the preceding 3D segment of the picture. The estimated tumor pixels' resulting values are as follows:

(14) Ep=Rv1/2hHRv1/2s|^2Rv1/2hHRv1/2 h,

(15) Ep=Rv1/2h^HRv1/2s^2Rv1/2h^HR1/2h≤πr2Rv1/2h^HR1/2hR−1/2s^HR−1/2sRv1/2h^HR1/2h,

(16) Ep=Rv1/2h2Rv1/2s2Rv1/2h^HRv1/2h≤sHRv−1s,

(17) $$\begin{array}{c} R^{1/2}h=aR^{-1/2}s, \end{array}$$

(18) $$\begin{array}{c} E_{p}=\frac{{\left|{\left(R_{v}^{1/2}h\right)}^{H}\left(R_{v}^{-1/2}s\right)\right|}^{2}}{{\left(R_{v}^{1/2}h\right)}^{H}\left(R_{v}^{1/2}h\right)}=\frac{\alpha^{2}{\left|{\left(R_{v}^{-1/2}s\right)}^{H}\left(R_{v}^{-1/2}s\right)\right|}^{2}}{\alpha^{2}{\left(R_{v}^{-1/2}s\right)}^{H}\left(R_{v}^{-1/2}s\right)}=\frac{{\left|s^{H}R_{v}^{-1}s\right|}^{2}}{s^{H}R_{v}^{-1}s}=s^{H}R_{v}^{-1}s. \end{array}$$

After passing images from the above equations, the image is passed through the following neural network to get segmented.

**Figure 1:**
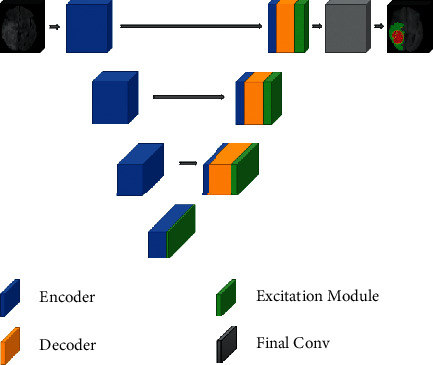
Proposed system architecture for the segmentation of brain tumor.

The three-dimensional attention module is merged with the decoding blocks, and the U-Net architecture is translated to 3D. A 3D attention model with decoder blocks is also available to improve segmentation estimation. A channel plus spatial attention system, as well as a bypass connection, makes up the attention module we propose. Combining concurrently intriguing features, on the other hand, may lead to pattern training inconsistency. When skip connections are employed, the network's redundancy and sparsity are lowered.  Figure 1 depicts the situation of projected structure architecture for segmentation of brain tumor.

Here the parallel and serial connection of block of encoder and decoder is presented. The encoder block encodes the result of the mathematical model (from equation (18)) and the left side of the U type structure takes it into account so that the size of the 3D block converges. The right side of the U type structure is the combination of encoder, decoder, and excitation module. After that, there is a block called convolution which convolves the result and gives the segmented result.

Spatial and channel attention improves encoding quality across the feature hierarchy. As a consequence, we create 3D concentration units that provide 3D spatial plus channel attention by combining 3D trans and cross feature interactions. To create the 3D attention map, we first combine all three-dimensional attribute connections focused on the H  ×  W  ×  1 measurement with a 1  × 1  ×  C convolution. We do parallel average pooling and forward it to the neural network to obtain the 1 × 1  ×  C channel correlation. Rich spatial and channel attention is stored in the 3D attention map. We also employ skip connection to decrease the sparsity and singularity that these parallel excitations create. Furthermore, using a skip connection broadens the learning and improves segmentation prediction.

## 3. Results and Discussion

All of the tests in the paper are done with BraTS 2019. BraTS 2019 has 335 cases, including 259 instances of high-grade glioma and 76 cases of low-grade glioma, respectively. In the validation and test sets, there are 125 and 166 examples, respectively. The modality has a voxel size of 240 × 240  ×  155. In the training set, there is also a segmentation annotation that marks three areas as 1, 3, and 4 pixel values.

**Figure 2:**
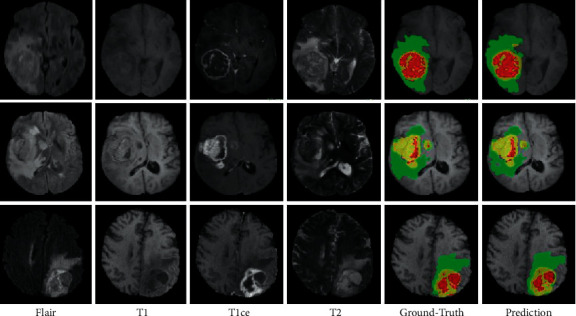
The Flair, T1, T1ce, and T2 channels of the brain tumor were displayed using the ground-truth and expected segments of tumor post using the BraTS 2019 cross validation dataset.

**Figure 3:**
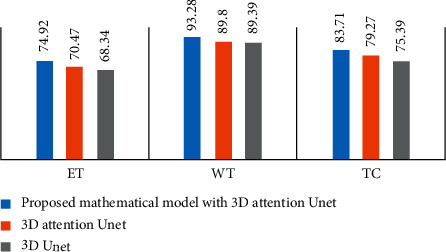
Comparison of the suggested mathematical model with 3D attention U-Net, 3D attention U-Net model, and the original 3D U-Net model in terms of performance.

We upload our estimation accuracy to the BraTS 2019 site and obtain a variety of measurement metrics to evaluate our model prediction, including Dice, Hausdorff, sensitivity, and specificity.  Table 1 presents the statistical parameters calculated from the BraTS 2019 validation set's performance parameters. A visual depiction of the validation set prediction is shown in Figure 2. Necrosis is shown by red colour, tumor enhancement is shown by yellow colour, and edema is shown by green colour. The presentation graphs of the suggested three-dimensional attention U-Net vs the original 3D U-Net are shown in Figure 3.

Figure 3 shows that the suggested model beats the 3D attentive U-Net and the 3D digital U-Net paradigm across all areas, including ET, WT, and TC.

**Figure 4:**
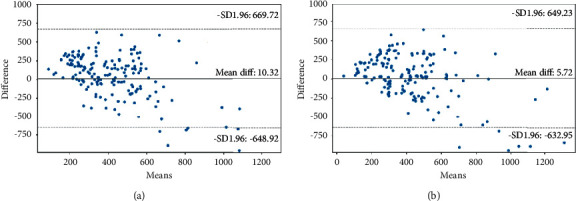
Bland–Altman plot generated from overall survival prediction model training cross validation results. (a) For all retrieved characteristics, a Bland–Altman plot was obtained. As a result, the average difference is 10.32 days. (b) The Bland–Altman plot for the 14 characteristics chosen. This results in a 5.72-day average difference.

To develop an effective model, we chose the 14 very vital properties and trained the models. A Bland–Altman plot in Figure 4 depicts the circulation of reversion productivity for entirely retrieved structures and 14 nominated attributes (Figures 4(a) and 4(b)). Compared to all other variables, the average gap between actual survival and anticipated survival rate for the selected attributes is nearly half (5.72 days).

**Figure 5:**
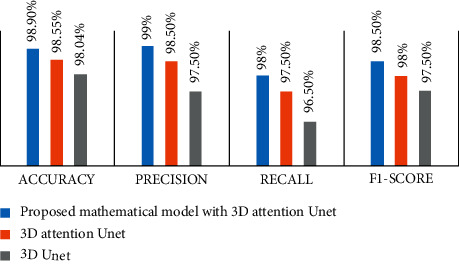
Pixel level performance parameters for the segmentation of tumor pixels.

Figure 5 clearly indicates that the suggested structure architecture is performing extraordinarily superior than other present system architectures. The accuracy, precision, recall, and F1 score of the recommended system architecture for segmentation are 99.90%, 99.90%, 98.50%, and 98.50%, respectively, which are much higher than those of existing systems.

**Figure 6:**
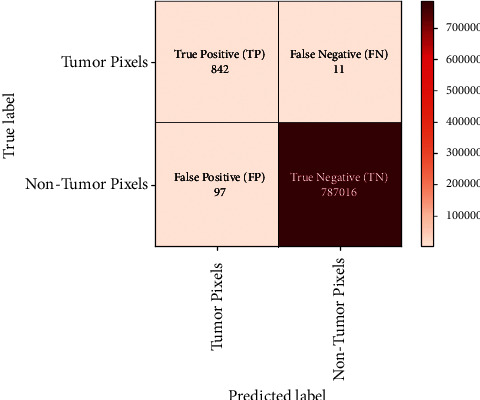
Confusion matrix of result of segmentation for pixel level.

When the results are compared on the basis of pixel value, then the resultant confusion matrix is presented in Figure 6. It clearly states that the dominant diagonal is having higher values than those of the other cells in the confusion matrix. The MRI images of the width 809 pixels and height 974 pixels are considered in the present study. The average result of 100 images is calculated and put in the form of confusion matrix here.

Here after taking average of 100 tumor images, the average tumor pixels are 853 and average non-tumor pixels are 787113.

The time complexity is tested on several CPUs. The average time it takes to get a outcome on several hardware platforms is shown in Table 2.

When using a CPU such as i5 or i7, the time complexity is approximately identical; however, when the system is evaluated on a GPU, the time necessary to get the results is much different.

## 4. Conclusion

BraTS 2019 was used to complete all of the experiments in the article. BraTS 2019 has 335 cases, with 259 instances of high-grade glioma and 76 cases of low-grade glioma. To test our model prediction, we upload our estimation accuracy to the BraTS 2019 site and obtain a number of measurement metrics, such as Hausdorff, sensitivity, Dice, and specificity. The suggested system architecture produces accurate tumor pixel segmentation findings from a 3D brain picture. The system is put to the test on a variety of levels. The types of feature extraction are utilized as the initial criterion. The temporal complexity of the systems is compared. The suggested system is also evaluated in comparison to current classifiers. The suggested system architecture for segmentation has accuracy, precision, recall, and F1 score of 99.90%, 99.90%, 98.5%, and 98.50%, respectively, which are significantly higher than those of existing systems. From this, we can conclude that the proposed system architecture is reliable and we can use it in the medical field for effective diagnosis.

## Figures and Tables

**Table 1:** The Dice, Hausdorff, sensitivity, and specificity measures were used to analyze the statistical characteristics of the BraTS 2019 testing dataset for the classification job.

	Dice			Hausdorff			Sensitivity			Specificity		
	ET	WT	TC	ET	WT	TC	ET	WT	TC	ET	WT	TC
Mean	0.603	0.823	0.712	6.92	6.24	8.53	0.723	0.895	0.793	0.934	0.852	0.721
StdDev	0.293	0.062	0.132	12.84	10.73	12.34	0.273	0.042	0.176	0.002	0.004	0.004
Median	0.724	0.823	0.821	2.21	3.17	4.17	0.824	0.835	0.843	0.834	0.926	0.926

**Table 2:** The time required to obtain the desired outcome utilizing the suggested system design on various hardware systems.

Platform	The time it takes to acquire a result (s)
8 GB RAM, CPU, i3 processor	0.724
8 GB RAM, CPU, i5 processor	0.532
8 GB RAM, CPU, i7 processor	0.518
Nvidia K80, GPU	0.009

## Data Availability

The data used to support the findings of this study are available from the corresponding author upon request.
